# Surgical ventricular restoration in patients LV aneurisms and congestive heart failure

**DOI:** 10.1186/1749-8090-8-S1-O168

**Published:** 2013-09-11

**Authors:** B Baev, D Petkov, R Iliev, D Kiuchukov, G Nachev

**Affiliations:** 1 Department of Cardiac Surgery, ‘St. Ekaterina’ University Hospital, Sofia, Bulgaria

## Background

SVR is increasingly performed in patients with LV aneurisms and CHF. We report our ten year results with different surgical reconstructions and two different methods of myocardial protection.

## Methods

Between January 2003 and January 2013, 390 consecutive patients underwent SVR, of whom 219 with ICHF, NYHA- class III- IV and EF< 35% were selected for this study. The patients are divided in two groups: Group I -113 pts with vented fibrillating heart, Group II –106 pts with cardioplegic arrest (ante/retrograde cold blood cardioplegia). The patients in both groups have similar pre- and intra-operative parameters. Euroscore averaged 11,2 (6,8- 57,9). Two surgical techniques were used: Dor procedure in 119 patients and linear resection in 100 patients. Concomitant to SVR, mitral valve reconstructions, LV septoplasty, CABG and trombectomy have been performed in 44 (20%), 28 (12,3%), 215 (98,1%) and 71 (32,4%) patients, respectively.

## Results

Mean follow-up was 57 ± 21 months. The operative mortality was 8,2% (18 pts). In both groups EF improved from 29% to 36,6%, NYHA-class was reduced from 3,3 to 1,5, LVEDV was reduced by 62 ± 43 ml and LVESV- by 55 ± 47 ml post-operatively. Significant differences between Group I and Group II were found in the use of inotropes 60% versus 84%, use of IABP 25,4% versus 43,4%, duration of mechanical ventilation 23,6 versus 47,9 hours and length of hospital stay 8,8 versus 12,8 days. No difference was found between the Dor procedure and linear resection in terms of mortality, NYHA class and LV volume reduction.

## Conclusions

SVR shows good results for the treatment of patients with ICHF and EF< 35%. The method of vented fibrillating heart showed lower occurrence of LCO and significantly shorter duration of mechanical ventilation and length of hospital stay in comparison to the method of cardioplegic arrest.

